# Effects of borneol on apoptosis of hypoxia/reoxygenation H9c2 cells and myocardial ischemia-reperfusion injury rats

**DOI:** 10.1590/acb402225

**Published:** 2025-03-10

**Authors:** Hui Zhang, Junfang Dong, Jianwu Zhang, Hongxue Chen, Ting Liu, Ruixue Gan, Jing Wen, Yangyou Li

**Affiliations:** 1 North Sichuan Medical College – Department of Pharmacy – Nanchong, Sichuan – China.; 2 North Sichuan Medical College – Animal Experimental Center – Nanchong, Sichuan – China.

**Keywords:** Reperfusion Injury, Myocardial Infarction, Apoptosis

## Abstract

**Purpose:**

To explore the protective effects of borneol in myocardial ischemia-reperfusion injury (MIRI) and the mechanism of apoptosis.

**Methods:**

Cell viability was detected by CCK-8. The total superoxide dismutase (T-SOD) and lactate dehydrogenase (LDH) leakage of cells were tested by biochemical assay kit. Detection of apoptosis was by flow cytometry. Serum levels of creatine kinase isoenzyme MB (CK-MB), LDH, and cardiac troponin I (cTnI) were detected by enzyme-linked immunosorbent assay. Myocardial infarction area and pathological changes were observed via 2,3,5-triphenyltetrazolium chloride (TTC) staining and hematoxylin and eosin staining. The expressions of apoptosis-related proteins in cells and myocardial tissues were detected by Western blot.

**Results:**

H9c2 cell viability was significantly increased by pretreatment with 16 and 32 μg/mL of borneol. Borneol pretreatment significantly increased the T-SOD levels and reduced LDH leakage and apoptosis. In MIRI rats, borneol pretreatment significantly reduced serum levels of CK-MB, LDH and cTnI, decreased myocardial infarction area, and improved myocardial injury in different degree. Western blot results showed that borneol pretreatment significantly reduced the expression of Bcl-2-associated X protein (Bax) and Cysteine-aspartate protease-3 (Caspase-3) in cells and myocardial tissues of rats.

**Conclusion:**

Borneol can protect myocardial injury cells and mitigate MIRI by inhibiting cardiomyocyte apoptosis.

## Introduction

Among cardiovascular diseases, ischemic heart disease is one of the leading causes of death and disability worldwide. In China, the incidence, disability and mortality of ischemic heart disease such as acute myocardial infarction (AMI) are on the rise, and the disease is getting younger and younger^1^. After the occurrence of ischemic cardiomyopathy such as AMI, the timely restoration of the blood supply is the key to rescuing the patient and protecting the cardiomyocytes. With the application of coronary artery bypass graft and percutaneous coronary intervention in clinical practice, myocardial injury due to rapid myocardial recanalization of ischemia (myocardial ischemia-reperfusion–MIRI) further deteriorates the cardiac function of the patients, while rescuing the patients and leads to poor therapeutic effects^2^. Therefore, it is of great significance to explore the molecular mechanism of MIRI and find effective drugs to prevent it.

Apoptosis is a programmed cell death process that accompanies by morphological changes and is considered to be the only type of cell death that is regulated^3^^,^ 4. After the occurrence of MIRI, apoptosis can be initiated by a variety of stimuli, such as hypoxia and energy deficiency, oxidative stress, deoxyribonucleic acid (DNA) damage, etc. After the initiation of apoptosis, various apoptotic pathways can be activated, and the activation of apoptosis is involved in the entire process of MIRI^5^. Excessive cell death is a key factor in the pathologic damage of myocardial infarction. Therefore, effective inhibition of excessive apoptosis is one of the research directions to alleviate MIRI.

Borneol is a terpene compound, which can be obtained by artificial synthesis or natural product extraction. Natural borneol can be extracted from the fresh branches and leaves of the *Cinnamomum camphora* 6. The extract of *C. camphora* (natural borneol) is a precious traditional Chinese medicine with the effects of inducing resuscitation, relieving pain, antibacterial and anti-inflammatory, etc. It is an important drug for first aid and treatment of neurological diseases in traditional medicine^7^^–^ 12. Modern pharmacological studies have demonstrated that borneol is anti-inflammatory^13^, inhibition of viruses^14^, and promote drug distribution and absorption^15^^,^ 16. As one of the active ingredients of traditional Chinese medicine coronary cardiotonic capsules, borneol has cardioprotective effects against MIRI and calcium overload inhibition^17^. The proprietary Chinese medicine Suxiao Jiuxin Pill, which contains borneol, has been also shown to reduce MIRI in rats^18^. Therefore, we speculate that borneol has a protective effect on MIRI.

In this study, we used hypoxia/reoxygenation (H/R) H9c2 rat cardiomyocyte model to simulate the clinical MIRI and explore the protective effects of borneol on H/R induced cardiomyocyte injury and its mechanism related to inhibition of apoptosis.

## Methods

### Cell culture and establishment of hypoxia/reoxygenation model

H9c2 rat cardiomyocytes (Procell, Wuhan, China) were cultured in DMEM high glucose medium (Boster, California, United States of America) containing 10% fetal bovine serum (Every green, Huzhou, China), 1% penicillin/streptomycin (Boster, California, United States of America) and in a constant temperature incubator with 5% CO_2_ at 37°C. Hypoxia was induced by culturing H9c2 cells in DMEM without glucose or serum in an atmosphere of 100% N2 for 5 h. Following hypoxia treatment, the H9c2 cells were returned to normoxic conditions (air with 5% CO2) for 24 h.

### Cell viability assay

H9c2 rat cardiomyocytes were divided into the following five groups:

- Control group;
- H/R group;
- H/R + 8: borneol (8 μg/mL) group;
- H/R + 16: borneol (16 μg/mL) group;
- H/R + 32: borneol (32 μg/mL) group.

H9c2 cells were pretreated with 8, 16, and 32 μg/mL borneol for 12 h before H/R treatment. H9c2 cells without borneol or H/R treatment served as a control. The viability of H9c2 cells was measured by a CCK-8 cell viability assay.

### Determination of superoxide dismutase in cells and lactate dehydrogenase content in culture medium

H9c2 cells were divided into five groups, as mentioned above. The total superoxide dismutase (T-SOD) (Njjcbio, Nanjing, China) content was measured after the cells were collected and homogenized by kits. Cell culture medium was collected, and lactate dehydrogenase (LDH) (Njjcbio, Nanjing, China) content in culture medium was detected by kits.

### Flow cytometry assay

Cell apoptosis was detected by the Annexin V-FITC/PI fluorescence double-staining apoptosis assay kit (Beyotime, Shanghai, China) with flow cytometry. H9c2 rat cardiomyocytes were divided into five groups, as mentioned above. The cells from each group were washed with phosphate buffered saline (PBS) twice and incubated with trypsin at 37°C for 2 min. Following digestion, the cell suspension was centrifuged at 3,000 r/min at room temperature for 5 min. The cell pellet was resuspended with PBS, and the centrifugation and resuspension steps were repeated twice. The cells were blocked with 10% bovine serum albumin for 30 min at room temperature. Before the test, incubate was performed with annexin V/PI dye solution for 10 min at room temperature in the dark. The apoptosis rate was determined by the annexin V-FITC apoptosis detection kit.

### Animal grouping and drug administration

In total, 105 male Sprague Dawley rats weighing 220 ± 10 g were obtained from the Laboratory Animal Center of North Sichuan Medical College (Nanchong, Sichuan, China), license number: SCXK (Jing) 2019-0010. The use of laboratory animals was approved by the Ethics Committee of North Sichuan Medical College (NSMC: 2022-04). The rats were placed under standard laboratory conditions (24°C ± 2°C, 60–70% humidity, 12-h light-dark cycle) with free food and water. After acclimatization, the rats were randomly divided into the following seven groups:

- Sham group (n = 15);
- MIRI model group (n = 15);
- Tween80 group (5%) (n = 15);
- Ramipril group (1 mg/kg, n = 15);
- Borneol 0.5 mg/kg group (n = 15);
- Borneol 1 mg/kg group (n = 15);
- Borneol 2 mg/kg group (n = 15).

The rats in each group were pretreated with the corresponding drug by intragastric administration for three days. For the last administration of drugs for 30 min, a myocardial infarction operation was performed.

### Myocardial ischemia-reperfusion injury models

After anesthesia with 25% urethane, the rats were connected to a ventilator to maintain respiration and connected to an electrocardiograph to record the rat electrogastrogram (ECG). The left chest was open to expose the heart, the pericardium was peeled off to find the coronary artery, and the anterior descending branch of the left coronary artery was ligated. The sham group was not ligated, and the same was treated. The MIRI model was established by 40 min of ischemia and 2 h of reperfusion. Ischemic success was judged by the elevation of the ST segment on the ECG. After reperfusion, blood serum and myocardial tissues were collected for further determination.

### Enzyme-linked immunosorbent assay assay

The content of serum creatine kinase isozyme (CK-MB) (Elabscience, Wuhan, China), Cardiac troponin I (cTnI) (Elabscience, Wuhan, China), and LDH (Elabscience, Wuhan, China) were tested in each group by using collected blood serum, and the experimental method steps were carried out strictly according to the kit instructions.

### TTC staining

The removed fresh heart tissue was quickly frozen, cut into five slices and stained with 1% 2,3,5-triphenyltetrazole chloride (TTC) (Nanjing Jiancheng Technology Co., Nanjing, China) for 15 min at 37°C. The infarct area was identified by the white color area, and the viable tissue was recognized as the red coloration. At last, the Image J software was employed to assess the infarct size. The calculation formula is as Eq. 1:


Myocardial infarction area(%)=White area / Total area of myocardial slice×100% (1)

### Hematoxylin-eosin staining

After reperfusion for 2 h, the ischemic ventricular tissue was taken to perform the hematoxylin-eosin (HE) staining. The tissue was cleaned with normal saline, fixed in 10% formalin buffer, dehydrated, paraffin-embedded, and then cut into slices about 4-mm thick. The slices were stained with a HE (Solarbio, Beijing, China) solution. Histopathological examination and photographs were taken to analyze the pathological changes of myocardial tissue in each group.

### Western Blot assay

H9c2 cells were divided into three groups: control group, H/R group, H/R + borneol group; tissues were divided into five groups: sham group, MIRI model group, borneol group, ramipril group, and Tween80 group. Standard Western blotting procedures were used. Antibodies used for Western blots include anti-Bax (Proteintech, Wuhan, China), anti-Caspase-3 (Proteintech, Wuhan, China), anti-β-actin (Proteintech, Wuhan, China) and anti-GAPDH (Proteintech, Wuhan, China) for Western blot.

### Statistical analysis

The data were expressed as mean ± standard deviation. Statistical Package for the Social Sciences 22.0 software and GraphPad Prism 9.0 were used for data analysis and statistical processing. One-way analysis of variance was used for comparison between groups. A value of p < 0.05 was considered statistically significant.

## Results

### Effects of borneol on cell viability and damage after hypoxia/reoxygenation modeling

**Figure 1:**
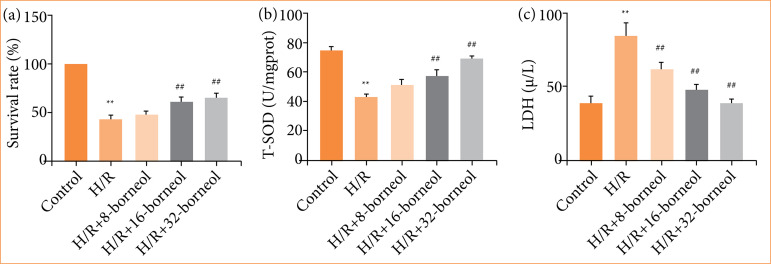
Effects of borneol on cell viability and damage after hypoxia/reoxygenation (H/R) modeling. H9c2 rat cardiomyocytes were pre-treated with 8, 16, 32 μg/mL borneol for 12 h before H/R treatment. **(a)** The viability of H9c2 cells; **(b)** content of total superoxide dismutase (T-SOD) in each group; **(c)** content of lactate dehydrogenase (LDH) in the cell culture medium of each group. Values were expressed as the mean ± standard deviation (n = 9).

After 5 h of hypoxia and 24 h of reoxygenation, cell viability was significantly decreased in the H/R group, and the cell viability increased with the concentration of the administered drug after borneol administration (Fig. 1a). In addition, the SOD content in the cells decreased significantly after H/R modeling, and the LDH content in the culture medium increased significantly, indicating that the cells were damaged during the H/R process (Figs. 1b and 1c). SOD content increased and LDH content decreased after borneol preadministration, suggesting that borneol were able to protect cardiomyocytes.

**p < 0.01 vs. control group; ##*p* < 0.01 vs. H/R group. Source: Elaborated by the authors.

### Effects of borneol on cell apoptosis and preliminary mechanism investigation

**Figure 2:**
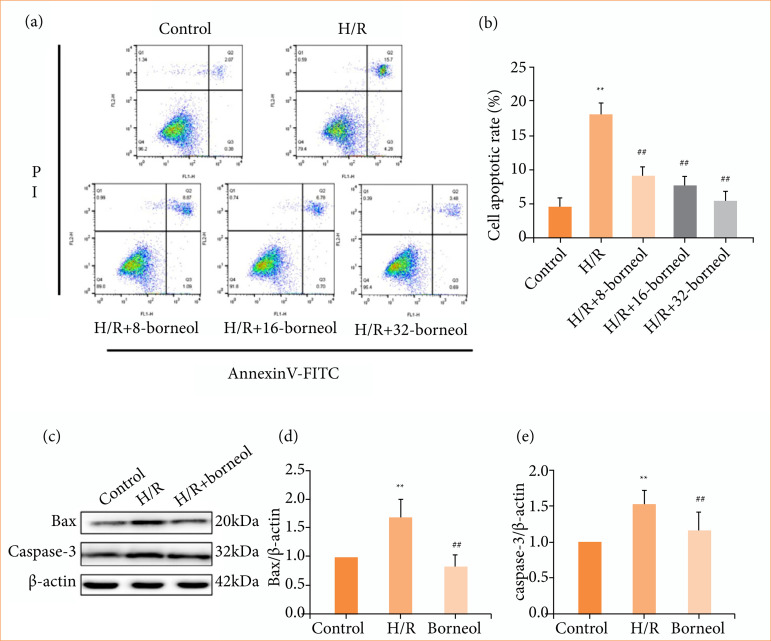
Effects of borneol on cell apoptosis and preliminary mechanism investigation. H9c2 rat cardiomyocytes were pre-treated with 8, 16, 32 μg/mL borneol for 12 h before hypoxia/reoxygenation (H/R) treatment. **(a)** Representative plot images of flow cytometry; **(b)** statistical analysis of cell apoptotic rate; **(c)** Western blots of Bax and Caspase-3 in H/R cells of each group; (**d** and **e**) mean relative level of Bax and Caspase-3 in H/R cells of each group. Values were expressed as the mean ± standard deviation (n = 8).

After 5 h of hypoxia and 24 h of reoxygenation, the number of apoptotic cells in H/R group was higher, and the rate of apoptosis was significantly increased compared with control group. The number of apoptosis was reduced after borneol pre-administration compared to H/R group. The results suggested that borneol might have the effect of inhibiting apoptosis in H/R cells (Figs. 2a and 2b). In addition, Western blot results showed that the expression of pro-apoptotic proteins Bax and caspase-3 was significantly increased in the H/R group compared with the control group, and the expression of both pro-apoptotic proteins was significantly decreased after the pre-dosing of borneol, which indicated that borneol might protect cardiomyocytes by inhibiting cell apoptosis (Figs. 2c–2e).

*p < 0.05; **p < 0.01 vs. control group; #*p* < 0.05; ##*p* < 0.01 vs. H/R group. Source: Elaborated by the authors.

### Effects of borneol on serum myocardial enzyme marker levels and histopathological changes of myocardial tissue

**Figure 3:**
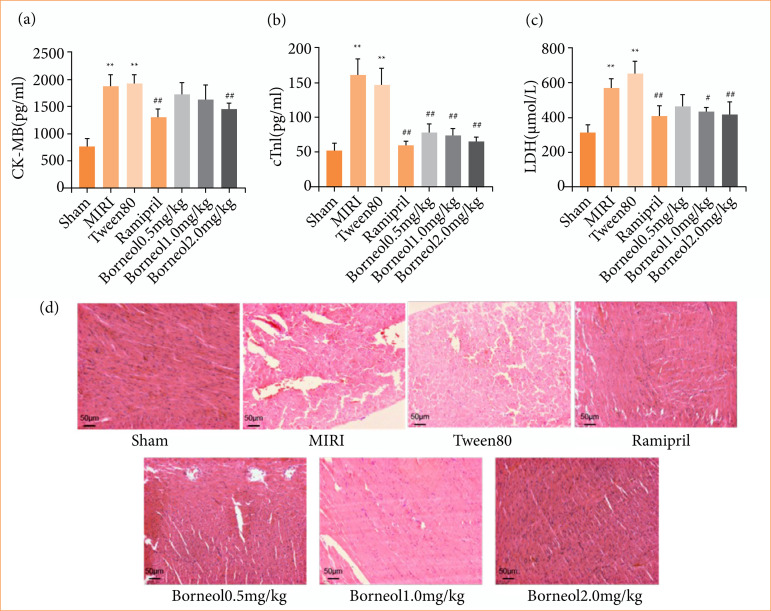
Effects of borneol on serum myocardial enzyme marker levels and pathological changes of myocardial tissue. **(a)** Enzyme-linked immunosorbent assay (ELISA) kit measures serum CK-MB content in rats; **(b)** ELISA kit measures serum cTnI content in rats; **(c)** ELISA kit measures serum lactate dehydrogenase (LDH) content in rats (n = 9); **(d)** the experimental results were visible under a 200× microscope (n = 5). Values were expressed as the mean ± standard deviation.

As shown in Fig. 3, CK-MB, cTnI and LDH were significantly higher in the MIRI and Tween80 groups compared with the sham group, indicating that the myocardium was damaged after ischemia-reperfusion. Compared with the Tween80 group, CK-MB was significantly lower in the borneol 2 mg/kg group (Fig. 3a), cTnI showed a gradual trend of decreasing with the increase in the dose of borneol administered (Fig. 3b), and LDH was lower in the 1 mg/kg group of borneol, while the trend of decreasing in the 2 mg/kg group was even more significant (Fig. 3c). HE staining showed that myocardial tissues of all groups except the sham group showed different degrees of injury, including myocardial tissue bleeding, interstitial vasodilation, and congestion, myocardial fibrolysis, lymphocyte infiltration, etc. The MIRI and Tween80 groups showed the most severe injury. Ramipril group and borneol administration group could ameliorate myocardial injury to different degrees, and myocardial tissue injury was gradually repaired with the increase of borneol (1 or 2 mg/kg) (Fig. 3d).

**p < 0.01 vs. sham group; #*p* < 0.05 vs. Tween80 group; ##*p* < 0.01 vs. Tween80 group; MIRI: myocardial ischemia-reperfusion injury. Source: Elaborated by the authors.

### Effect of borneol on infarct area and verification of apoptosis mechanism

**Figure 4:**
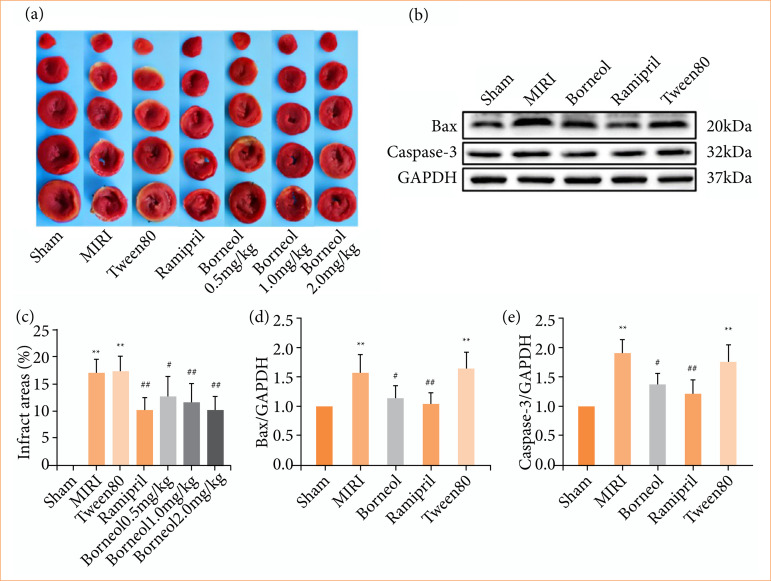
Effects of borneol on infarct area and verification of apoptosis mechanism. **(a)** Rat hearts were collected and stained with 2,3,5-triphenyltetrazole chloride. White indicated infarction, and red indicated normal tissue; **(b)** Western blots of Bax and Caspase-3 in cardiomyocytes of each group; **(c)** percentage of infarct area (n = 8); **(d)** mean relative level of Bax in cardiomyocytes of each group (n = 6); **(e)** mean relative level of Bax and Caspase-3 in cardiomyocytes of each group (n = 5). Values were expressed as the mean ± standard deviation.

The results of TTC staining showed that the myocardium of rats in the sham group was rose-red with no obvious infarcted area. There were obvious infarcted areas (pale) in both the MIRI group and the Tween80 group. Ramipril and borneol improved myocardial infarction to varying degrees (Fig. 4a). Myocardial infarction area was significantly reduced in ramipril and borneol administration of 1 and 2 mg/kg (Fig. 4b). To verify the relationship between the protective effect of borneol against MIRI and apoptosis, we further determined the expression levels of relevant apoptotic proteins in myocardial tissues. Western blot experiments showed that, in the MIRI rat model, the expression of Bax and Caspase-3 was increased in the MIRI group and the Tween80 group compared with the sham group. The expression of Bax and Caspase-3 was decreased in the borneol group compared with the Tween80 group (Figs. 4c–4e). The experimental results of myocardial tissues and H9c2 cells were consistent, suggesting that borneol may inhibit apoptosis to ameliorate MIRI.

**p < 0.01 vs. sham group; #p < 0.05 vs. Tween80 group; ##p < 0.01 vs. Tween80 group; MIRI: myocardial ischemia-reperfusion injury. Source: Elaborated by the authors.

## Discussion

To the best of our knowledge, I/R injury often occurs under pathological conditions with complex mechanisms^19^. It is widely recognized that apoptosis is one of the major forms of cell death in heart disease, and that cardiomyocyte apoptosis is an important determinant of irreversible damage caused by I/R. Borneol is a traditional Chinese medicine that has the effects of refreshing the mind, aromatizing, and promoting resuscitation, clearing heat, and dispersing toxins. It is often used clinically as a compatible drug in the treatment of angina pectoris and coronary heart disease and other cardiovascular diseases. Studies have shown that edaravone dexborneol (edaravone and (+) - borneol = 4:1) can improve neural function, inhibit neuronal cell apoptosis, which is more effective than edaravone alone^20^. L-borneol can stabilize mitochondrial homeostasis and inhibit apoptosis signals and neuronal apoptosis during the pMCAO process, thereby achieving brain protection^21^. This study aimed to investigate the mechanism by which borneol to protect H/R cells and MIRI rats by inhibiting apoptosis.

In the hypoxia environment, abnormal cellular oxidative metabolism processes produce many free radicals, which cause oxidative stress reaction, leading to DNA damage, lipid peroxidation, and protein oxidation^22^. The accumulation of excess oxygen free radicals triggers oxidative stress, leading to further damage. However, the antioxidant system in cells can neutralize free radicals, protect cells from oxygen free radicals and reduce cellular damage^23^^,^ 24. SOD protects cells from free radical damage. Spillage of LDH indicates impaired cellular energy metabolism or redox imbalance. Spilled LDH can damage membrane lipids by producing peroxides and damage. However, since whether LDH is stable or not can reflect the extent of cellular damage, increased permeability of the plasma membrane allows for rapid spillover of LDH, a key feature leading to cellular damage^25^. The results of this study showed that borneol could significantly increase the T-SOD level in cells and reduced LDH cellular spillover caused by H/R in H9c2 cells, suggesting that borneol alleviated H/R-induced injury.

Leakage of CK-MB and LDH from cardiomyocytes leads to elevated serum concentrations, which are markers of myocardial injury that reflect the degree of myocardial injury^26^. cTnI is the gold standard biomarker for detecting cardiac injury and necrosis of cardiomyocyte, and is highly specific for cardiac injury, and persistent elevation of troponin I is associated with greater left ventricular dysfunction and a higher rate of cardiac events^27^, which are combined with the myocardial infarction area to assess the degree of myocardial damage. The data showed that the above serum indexes and myocardial infarction area were significantly increased in the MIRI group, indicating that the degree of myocardial damage was aggravated, while the pretreatment of borneol was significantly reduced. In histopathological experiments, the results coincided with the above. Our results confirmed that borneol can reduce the extent of heart muscle damage.

Apoptosis is a major pathophysiology feature of early I/R and can lead to more severe heart failure. During the execution of apoptosis, the extramitochondrial membrane B-cell lymphoma 2 (Bcl-2) protein family located on the mitochondrial pathway regulates myocardial survival and mortality in MIRI^28^. The Bcl-2 protein family can be divided into pro-apoptotic proteins (Bax, Bad, and Bak) and anti-apoptotic proteins (Bcl-2, Bcl-xL, and Bcl-B) based on different structural domains, playing an important role in initiating the intrinsic apoptotic pathway^29^. Caspase is a family of cysteine proteases that play a crucial role in the apoptosis process of myocardial ischemia-reperfusion cells^30^. Ischemia-reperfusion injury stimulates Bax translocation into mitochondria, releasing proapoptotic proteins such as cytochrome C, thereby activating Caspase-3^31^. In a mouse model of myocardial ischemia-reperfusion, sinomenine and indole-3-methanol (I3C) protect the myocardium by inhibiting cardiomyocyte apoptosis by reducing the expression of Bax protein and Caspase-3^31^^,^ 32. Similarly, our data streaming cytometry analysis for *in-vitro* experiments showed a high rate of apoptosis in cardiomyocytes in the H/R group. In addition, the levels of pro-apoptotic proteins Bax and Caspase-3 were up-regulated in cardiomyocytes, and these results were verified in MIRI animal models. *In-vivo* and *in-vitro* borneol administration can significantly inhibit the expression levels of apoptosis and pro-apoptotic proteins Bax and Caspase-3.

No drugs have been found to treat MIRI with multiple targets. However, we have discovered an effective drug during apoptosis and initially revealed its possible mechanism, providing a new strategy for the treatment of MIRI.

## Conclusion

Borneol can protect the H/R H9c2 cells and MIRI rats, and its protective effect may be related to the inhibiting of apoptosis induced by H/R and MIRI. Therefore, borneol can be used as a preventive and therapeutic agent to reduce MIRI, and its effect on MIRI warrants further study.

## Data Availability

Data will be available upon request.
